# Associations of Oral Health-Related Quality of Life with age, oral status, and oral function among psychiatric inpatients in Japan: a cross-sectional study

**DOI:** 10.1186/s12903-020-01355-5

**Published:** 2020-12-14

**Authors:** Satoru Haresaku, Fuyuko Nakashima, Yayoi Hara, Madoka Kuroki, Hisae Aoki, Keiko Kubota, Toru Naito

**Affiliations:** 1 Department of Nursing, Fukuoka Nursing College, 2-15-1 Tamura, Sawara-ku, Fukuoka, 814-0193 Japan; 2 Department of Dental Hygiene, Fukuoka College of Health Sciences, 2-15-1 Tamura, Sawara-ku, Fukuoka, 814-0193 Japan; 3 Section of Geriatric Dentistry, Department of General Dentistry, Fukuoka Dental College, 2-15-1 Tamura, Sawara-ku, Fukuoka, 814-0193 Japan

**Keywords:** OHRQoL, Psychiatric disorders, Deglutition, Deglutition disorder, Oral diadochokinesis

## Abstract

**Background:**

As the general population of Japan ages, the population of hospitalized psychiatric patients is also ageing. The purpose of this study was to investigate the associations of oral health-related quality of life (OHRQoL) with age and oral health, including oral and swallowing function, among psychiatric inpatients.

**Methods:**

The subjects included 165 psychiatric inpatients in psychiatric hospitals in Japan. The General Oral Health Assessment Index (GOHAI) and the Eating Assessment Tool (EAT-10) were included in the questionnaire survey for the measurement of OHRQoL and the screening of dysphagia. A score ≥ 3 on the EAT-10 was defined as suspected dysphagia. Oral examinations and oral diadochokinesis (ODK) measurements for the tongue-lip motor function evaluation were conducted. The inpatients with acute psychiatric symptoms, moderate and severe dementia, and cognitive impairment that affected their ability to communicate and relate their feelings were excluded. A chi-squared test, the Mann–Whitney U test, and linear regression analysis were used for the analysis. The data were analysed at the 5% significance level.

**Results:**

A total of 100 (64.5%) psychiatric inpatients (mean age, 67.3 [SD, 14.5] years, 49% males, and 51% females) participated in this study. The means ± SDs for the decayed missing filled teeth (DMFT) index and GOHAI score were 20.6 ± 6 and 49.7 ± 7.9, respectively. The GOHAI score in the older age group (≥ 65 years) was significantly lower than that in the younger age group (< 65 years). The mean ODK scores were less than 3 times/s for all syllables. The percentage of the participants with suspected dysphagia was 45.0%. Tooth loss and suspected dysphagia were significantly associated with low GOHAI scores. The EAT-10 score was significantly correlated with the GOHAI score only after adjusting for age and sex (β = − 0.725, 95% CI − 0.97, − 0.64).

**Conclusions:**

In hospitalized psychiatric patients, impaired oral health in the older subjects was more pronounced compared with that among general adults. Tooth loss and swallowing function were associated with OHRQoL. Therefore, oral care for the recovery of occlusal and swallowing functions may be needed to improve OHRQoL among psychiatric patients.

## Background

The number of patients with psychiatric disorders is increasing worldwide. A report from the World Health Organization stated that the lifetime prevalence of psychiatric problems is approximately 20–35% [1, 2]. A survey conducted in Japan by the Ministry of Health, Labour and Welfare in 2017 estimated that 513 thousand patients (252 thousand inpatients and 261 thousand outpatients) were diagnosed with psychiatric and behavioural disorders [3]. With respect to the age of the psychiatric inpatients, the majority were 40 years and over, and the percentage of inpatients aged 65 and over increased considerably from 28.5% in 1999 to 58.4% in 2017 [3, 4].

Several original international studies [5–14] and one meta-analysis [15] reported that the oral health status of psychiatric patients was poor compared to that of the general population. In addition, some previous studies reported that patients with schizophrenia and dementia had poor swallowing function and were at risk for aspiration pneumonia [16, 17].

Oral health-related quality of life (OHRQoL) is an integral part of general health and well-being and is recognized by the WHO as an important segment of the Global Oral Health Program [18]. OHRQoL scales are used to assess a patient’s condition or a change in oral status during the course of care and to integrate the perceptions and expectations of the patient. The General Oral Health Assessment Index Questionnaire (GOHAI) has been widely used to assess oral health in clinical and epidemiological studies [19]. The GOHAI assesses self-perceived oral health through 12 questions that explore the pain, discomfort, dysfunctions and psychosocial impacts associated with dental diseases [20]. Only two studies using the GHOHAI for psychiatric patients reported that the OHRQoL score in psychiatric patients was lower than that in the general population [21, 22], and there have been no studies performed on psychiatric patients’ OHRQoL in Japan.

Several studies have reported that age, the number of remaining teeth, regular dental check-ups, and chewing function were associated with the GOHAI score [23–29]. Therefore, those factors might be associated with psychiatric patients’ OHRQoL. However, no studies have investigated the associations of OHRQoL with oral and swallowing functions among psychiatric patients.

The purpose of this study was to investigate oral health, including oral and swallowing function, and OHRQoL and the associations of OHRQoL with age and oral health among psychiatric inpatients.

## Methods

### Design and sample

This study was a cross-sectional survey of psychiatric inpatients in two psychiatric hospitals in Fukuoka Prefecture, Japan. Fukuoka Prefecture is situated on the northern shore of the Japanese island Kyushu. Hospital A was a prefectural hospital and had 300 beds. Hospital B was a medical corporation hospital and had 270 beds. The subjects were recruited in the chronic phase wards (4 wards in hospital A and 2 wards in hospital B). The total number of subjects was 165.

### Questionnaire survey

The questionnaire consisted of the following 3 parts: socio-demographic data, the GOHAI questionnaire for the measurement of OHRQoL [19], and the 10-Item Eating Assessment Tool (EAT-10) for the identification of swallowing problems and the screening of suspected dysphagia [30].

Socio-demographic information included sex and age, length of hospitalization, last psychiatric diagnosis (according to the International Classification of Diseases 10th Revision: ICD-10), and drug use for psychiatric disorders.

The Japanese version of the GOHAI was used in the questionnaire and was composed of 12 items [31, 32]. The 12 items assessed physical function (eating, talking and swallowing) in items 1, 2, 3 and 4 and psychosocial impacts (self-esteem, social withdrawal and worries about oral health) in items 6, 7, 9, 10 and 11. Items 5, 8 and 12 assessed pain and symptoms (use of drugs to relieve pain, discomfort) related to the presence of oral diseases. There are five response categories with an associated score (l = always, 2 = often, 3 = sometimes, 4 = seldom, and 5 = never). The GOHAI score is computed by summing the scores of the 12 responses, and the highest score (60) indicates excellent oral health.

The Japanese version of the EAT-10 [33] was used to identify swallowing problems and screen for suspected dysphagia. The EAT-10 consists of ten items regarding swallowing problems. Each question is scored from 0 (no problem) to 4 (severe problem). An elevated EAT-10 score indicates a severe risk of dysphagia. Participants were divided into two groups: those with an EAT-10 score between 0 and 2 and those with an EAT-10 score between 3 and 40 because a score ≥ 3 was defined as the prevalence of suspected dysphagia in previous studies [34, 35].

The validity and reliability of the Japanese questionnaires were verified in previous studies [31–33]. Cronbach’s alpha values for each domain ranged from 0.894 in the GOHAI to 0.942 in the EAT-10.

### Oral examinations

The clinical examinations were conducted in the wards by one dentist whose profession was preventive dentistry and who had more than 20 years of experience with dental examinations for research. He examined the participants with a mirror, a probe and a transillumination lamp without the use of radiographs. The participants sat on a chair during the examination. The clinical assessment was recorded according to the WHO criteria [36]: severity of lifetime accumulated caries estimated with the decayed-missing-filled teeth (DMFT) index [number of decayed teeth (DT), missing teeth due to decay (MT), and filled teeth (FT)].

### Oral diadochokinesis

Oral diadochokinesis (ODK) was used for the comprehensive measurement of the motor speed and dexterity of the tongue and lips. ODK has been used in older Japanese populations [37, 38]. After the oral examinations, the participants were instructed to say each of the syllables /pa/, /ta/, and /ka/ repeatedly for 5 s. Pronouncing the syllables /pa/, /ta/, and /ka/ involves the use of the front (lips), middle (tip and the tongue), and back of the mouth (posterior tongue), respectively. The number of respective syllables produced per second was determined using an automatic counter (Kenkokun Handy, Takei Scientific Instruments Co., Ltd.) [39]. A diagnosis of decreased tongue-lip motor function was made when the number of /pa/, /ta/, or /ka/ syllables said per second was less than 6.

### Data procedure

Before the investigations, the subjects with acute psychiatric symptoms and moderate-severe dementia were excluded by the nurses and doctors in charge of them (the first selection). Two investigators who were psychiatric nurses visited the hospitals to recruit and interview the subjects. The selected subjects were initially eligible to receive an explanation of the study and were interviewed with the GOHAI and EAT-10; those who could not understand the explanation of this study, communicate their feelings, or respond after a question was repeated twice were also excluded based on the judgement of the investigators and nurses in charge of these patients (the second selection). Socio-demographic information, such as sex, age, length of hospitalization, last psychiatric diagnosis, and drug use for psychiatric disorders, was retrieved from the institutional medical records, and the information was filled out in the questionnaire by the two investigators. After the questionnaire survey, oral examinations were performed by a dentist and ODK measurements were performed by the two investigators at the second visit. The questionnaire surveys were administered, and the socio-demographic information was confirmed in hospital A from June to July 2018 and in hospital B from November to December 2018. The oral examinations and the ODK measurements were conducted in August 2018 in hospital A and in September 2018 in hospital B.

### Ethics

This study was approved by the Ethics Committee of Fukuoka Gakuen, Fukuoka, Japan (approved #366) and was performed in accordance with the 1964 Declaration of Helsinki and its later amendments or comparable ethical standards. The study was explained, and written informed consent was obtained from the inpatients.

### Data analysis

A chi-squared test was used to explore the differences in nominal variables between age groups or GOHAI groups. The Mann–Whitney U test was used to explore the differences in the ordinal variables between age groups or between GOHAI groups. Spearman's rank correlation was used to explore the correlations between OHRQoL, EAT-10, and other variables. Linear regression was used to identify relationships between OHRQoL and variables after adjusting for sex and age. Missing data were excluded from the analysis. The data were analysed at the 5% significance level. The statistical analyses were performed using the IBM SPSS Statistics software program (version 21.0; IBM Corporation, Armonk, NY, USA).

## Results

Forty-two and 23 inpatients were excluded in the first and second selections, respectively. All patients participated in the oral examinations and the ODK measurements. Therefore, a total of 100 psychiatric inpatients (64.5%) participated in all the investigations.

The majority of the participants (51.0%) were female and the mean ± SD age was 67.3 ± 14.5. (Table 1). The two most common psychiatric disorders were schizophrenia (F20–29 in ICD-10; 46.0%) and dementia (F00–09; 27.0%). Total of 76 inpatients used major Tranquilizers and total of 85 inpatients used minor Tranquilizers. Twenty-two patients with dementia used dementia medications. The mean ± SD of the GOHAI score was 49.7 ± 7.9. The mean ± SD of the numbers of DT, MT, and FT were 1.7 ± 3.1, 11.1 ± 9.8, and 7.8 ± 6.5, respectively. The mean ± SD of the DMFT index was 20.6 ± 6.8. The mean ± SD of the ODK scores were as follows: /pa/: 2.9 ± 2.0/s; /ta/: 2.9 ± 2.0/s and; /ka/: 2.7 ± 1.8/s. The percentage of participants with suspected dysphagia (EAT-10 score ≥ 3) was 45.0%. Significant differences were found in the type of psychiatric disorder, the number of MT, the number of FT, the DMFT, /pa/, /ta/, /ka/ and the EAT-10 score between age groups (p < 0.05).

**Table 1:** Comparison of characteristics, OHrQOL, and other variables according to age group

	Total	< 65 y	≥ 65 y	P value
Sex [n (%)]				
Male	49 (49.0%)	20 (48.8%)	29 (49.2%)	0.971*
Female	51 (51.0%)	21 (51.2%)	30 (50.8%)	
Age				
Years (Mean ± SD)	67.3 ± 14.5	53.4 ± 9.2	77.0 ± 8.2	-
Length of hospitalization				
Years (Mean ± SD)	9.4 ± 11.5	10.0 ± 10.0	9.0 ± 12.5	0.072
(ICD code) Type of psychiatric disorder [n (%)]				
Schizophrenia (F20–F29)	46 (46.0%)	25 (61.0%)	21 (35.6%)	< 0.001*
Dementia (F00–F09)	27 (27.0%)	1 (2.4%)	26 (44.1%)	
Mood disorders (F31, F32)	9 (9.0%)	4 (9.8%)	5 (8.5%)	
Others	18 (18.0%)	11 (26.8%)	7 (11.9%)	
OHrQOL				
GOHAI score (Mean ± SD)	49.7 ± 7.9	51.1 ± 8.3	48.7 ± 7.5	0.120**
Number of decayed teeth				
DT (Mean ± SD)	1.7 ± 3.1	2.5 ± 4.2	1.2 ± 2.0	0.124**
Number of missing teeth				
MT (Mean ± SD)	11.1 ± 9.8	6.5 ± 7.2	14.3 ± 10.2	< 0.001**
Number of teeth with fillings				
FT (Mean ± SD)	7.8 ± 6.5	9.6 ± 6.7	6.6 ± 6.1	0.016**
Total number of DT, MT, and FT				
DMFT(Mean ± SD)	20.6 ± 6.8	18.6 ± 6.9	22.1 ± 6.5	0.011**
Oral diadochokinesis (ODK, times/s)				
/pa/ (Mean ± SD)	2.9 ± 2.0	3.9 ± 1.6	2.2 ± 2.0	< 0.001**
/ta/ (Mean ± SD)	2.9 ± 2.0	4.0 ± 1.6	2.2 ± 2.0	< 0.001**
/ka/ (Mean ± SD)	2.7 ± 1.8	3.4 ± 1.5	2.2 ± 1.9	0.002**
EAT-10 group [n (%)]				
0–2	55 (55.0%)	28 (68.3%)	27 (45.8%)	0.026*
≥ 3 (Suspected dysphagia)	45 (45.0%)	13 (31.7%)	32 (54.2%)	

*Chi-squared test

^**^Mann–Whitney U test

Table 2 shows the comparisons of the nominal variables according to the 50^th^ percentile of the GOHAI. No significant differences were found in sex or the types of psychiatric disorder between the groups. However, 74% of the participants with low GOHAI scores (< 50th percentile) had suspected dysphagia, and a significant difference was found in the EAT-10 score between the low and high GOHAI groups (p < 0.001).

**Table 2:** Comparison of nominal variables according to 50th percentile of GOHAI score group

Nominal variables	Total	50th percentile GOHAI score		P value*
		Low	High	
Sex [n (%)]				
Male	49 (49.0%)	26 (52.0%)	23 (46.0%)	0.550
Female	51 (51.0%)	24 (48.0%)	27 (54.0%)	
Type of psychiatric disorder (ICD) [n (%)]				
Schizophrenia (F20–F29)	46 (46.0%)	28 (56.0%)	18 (36.0%)	0.251
Dementia (F00–F09)	27 (27.0%)	11 (22.0%)	16 (32.0%)	
Mood disorders (F31, F32)	9 (9.0%)	4 (8.0%)	5 (10.0%)	
Others	18 (18.0%)	7 (14.0%)	11 (22.0%)	
EAT-10 group [n (%)]				
0–2	55 (55.0%)	13 (26.0%)	42 (84.0%)	< 0.001
≥ 3 (Suspected dysphagia)	45 (45.0%)	37 (74.0%)	8 (16.0%)	

^*^Chi-squared test

Table 3 shows the comparisons of the ordinal variables according to the percentiles (25th, 50th, and 75th) of the GOHAI score. No significant differences were found in the medians of the number of DT, the number of FT, the DMFT index, or the oral ODK scores between the low and high GOHAI groups in all three percentile categories. A significant difference was found in the median of the number of DT between the GOHAI score < 25th percentile group and the GOHAI score ≥ 25th percentile group (p < 0.05), and a significant difference was found in the medians of the EAT-10 score between the low and high GOHAI score groups in all three percentile categories (p < 0.001).

**Table 3:** Comparison of ordinal variables according to the 25th, 50th, and 75th percentiles of GOHAI score

Category	25th percentile of GOHAI score			50th percentile of GOHAI score			75th percentile of GOHAI score		
Group	Median			Median			Median		
Variables	Low	Low	P value*	Low	Low	P value*	Mean rank	Mean rank	P value*
DT	21.0	24.5	0.234	21.0	22.5	0.513	19.0	23.0	0.177
MT	1.0	0.0	0.204	1.0	0.0	0.102	1.0	0.0	0.019
FT	8.5	10.0	0.639	6.5	10.0	0.355	4.0	10.0	0.161
DMFT	8.0	7.0	0.172	8.5	7.0	0.334	9.5	7.0	0.107
ODK									
/pa/	3.2	3.6	0.601	3.4	3.3	0.325	3.4	3.3	0.856
/ta/	3.2	3.7	0.831	3.0	3.6	0.854	3.0	3.6	0.789
/ka/	3.2	3.1	0.713	3.2	3.1	0.479	3.1	3.2	0.885
EAT-10 score	0.0	15.5	< 0.001	0.0	10.0	< 0.001	0.0	7.0	< 0.001

^*^Mann–Whitney U test

Table 4 shows the correlations between OHRQoL, EAT-10, and other variables. Age, the length of hospitalization, the number of MT, the DMFT index, and the number of /Ka/ syllable repetitions per second were significantly correlated with the EAT-10 score. The number of MT and the EAT-10 score were significantly correlated with the GOHAI score (Spearman correlation coefficient = − 0.218 in DT and − 0.686 in EAT-10). After adjusting for sex and age, only the EAT-10 score was significantly correlated with the GOHAI score (β = − 0.725, 95% confidence interval: − 0.97, − 0.64, and p < 0.001).

**Table 4:** Correlations between EAT-10, OHrQOL, and other risk factors

	EAT-10		GOHAI	
	Correlation coefficient	p*	Correlation coefficient	p*
GOHAI	− 0.686	< 0.001	1.000	–
EAT-10	1.000	–	− 0.686	< 0.001
Age	0.239	0.017	− 0.070	0.487
Length of hospitalization	− 0.230	0.021	0.166	0.098
DT	− 0.089	0.376	0.123	0.222
MT	0.307	0.002	− 0.218	0.029
FT	− 0.162	0.107	0.111	0.270
DMFT	0.256	0.010	− 0.162	0.108
/Pa/	− 0.183	0.069	0.003	0.980
/Ta/	− 0.129	0.202	− 0.066	0.512
/Ka/	− 0.209	0.037	0.041	0.686

^*^Spearman's rank correlation

Table 5 shows the percentages of having swallowing problems and the medians of GOHAI score according to whether they had the problem in EAT-10 items. Approximately 20–40% inpatients had the problems regarding swallowing in the items. There were significant differences were found in GOHAI scores between having no problem and having the problem in all EAT-10 items.

**Table 5:** The percentages of having swallowing problems and the median of GOHAI score according to having the swallowing problem in EAT-10 items

	Having problem		GOHAI score (median)	
	%	Having problem	Having no problem	P value*
My swallowing problem has caused me to lose weight	32.0	44.5	54.0	< 0.001
My swallowing problem interferes with my ability to go out for meals	24.0	44.0	53.0	< 0.001
Swallowing liquids takes extra effort	32.0	45.0	54.0	< 0.001
Swallowing solids takes extra effort	38.0	45.0	55.0	< 0.001
Swallowing pills takes extra effort	40.0	45.0	55.0	< 0.001
Swallowing is painful	37.0	45.0	55.0	< 0.001
The pleasure of eating is affected by my swallowing	41.0	45.0	55.0	< 0.001
When I swallow food sticks in my throat	44.0	45.0	54.0	< 0.001
I cough when I eat	43.0	45.0	55.0	< 0.001
Swallowing is stressful	37.0	45.0	54.0	< 0.001

^*^Mann–Whitney U test

## Discussion

This report is the first to investigate the associations between age, OHRQoL and oral health, including oral function, among psychiatric inpatients. A previous study in France reported that the mean ± SD of the GOHAI score was 45.5 ± 8.4 among schizophrenic patients and that the GOHAI score was lower than that of the general population [21]. A previous study in Japan reported that the mean GOHAI scores among general Japanese adults (20–69 years) ranged from 51.3 to 54.8 [23]. Therefore, the results of this study showed that the OHRQoL among Japanese psychiatric inpatients was lower than that among the general Japanese population, and the tendency was similar to that in other countries. With respect to oral health, a Japanese national survey in 2016 reported that DT and MT in the general Japanese population (≥ 65 years) were 0.8 and 9.7, respectively [40]. Therefore, the oral health status of the psychiatric inpatients was poorer than that of the general Japanese population.

The results of this study showed that the number of syllable repetitions per second among the inpatients were nearly 3.0, which were much lower than the standard value. A previous study reported that older individuals are weaker than younger individuals, exhibit reduced force control and have slower neuromuscular contractile properties; therefore, ageing might contribute to reduced tongue motor function, as reflected in fewer repetitions [41]. Some previous studies reported that dementia [42], schizophrenia [43], and antipsychotic medication [43, 44] were associated with reduced motor function. In addition to those risk factors, some subjects repeated slowly the syllables although they were instructed to do as quickly as possible. It might be difficult for them to repeat quickly the syllables as instructed due to the low cognitive function. Therefore, it is suggested that the factors of the low number of the repetitions among the psychiatric patients might be not only low tongue mortar function but also low cognitive functions in psychiatric diseases. Further studies are needed to evaluate accurately the ability of tongue motor function for psychiatric patients.

With respect to swallowing function, the inpatients had a high prevalence of suspected dysphagia and 20–40% had the swallowing problems. A previous study for elderly people reported that fewer number of tooth and impaired tongue motor function were associated with lower swallowing function [45, 46]. Swallowing problems due to aging were more likely to develop in individuals with fewer teeth [45]. The number of teeth and tongue are important for forming a bolus with a viscosity and particle size suitable for swallowing without a delay in the initiation of swallowing [46]. Therefore, the low oral functions among the inpatients might impaired swallowing function and contribute to having their swallowing problems such as taking effort for swallowing solids or swallowing pain.

In addition to those swallowing problems, they had problems that they took extra efforts for swallowing liquid, pills, or sticky food. Those problems were thought to be caused by swallowing function itself. A previous study reported that ligamentous laxity, reduced muscle tone in the pharynx and esophagus, and increased duration of swallowing were recognized as physiological changes due to aging [46]. Patients with Alzheimer’s dementia tended to have an increased number of swallows for any given amount in their mouth, a longer duration of the swallow, and a longer period of apnea [47]. They developed reduced pharyngeal clearance, reduced upper esophageal opening, and penetration and/or aspiration as the disease progresses [48]. Some epidemiological studies reported that dementia was one of the main risk factors for dysphagia and aspiration pneumonia [17, 49]. Moreover, the prevalence of dysphagia was high among schizophrenic patients due to the nature of schizophrenia and the side effects of antipsychotic medication [44, 50, 51]. Therefore, factors such as aging, psychiatric diseases, and psychiatric medications might affect the swallowing problems among the inpatients. Dysphagia significantly worsens patient quality of life. It also results in increased morbidity and mortality, mainly due to a higher risk of aspiration pneumonia [52]. Therefore, further studies of dysphagia in psychiatric patients are needed.

Suspected dysphagia was associated with a low GOHAI score at the bivariate level. In addition, only the EAT-10 score was correlated with the GOHAI score after adjusting for sex and age. Having swallowing problems were significantly associated with low OHRQoL. It was hypothesized that OHRQoL were significantly associated with poor oral health status among psychiatric patients. However, our study showed that their OHRQoL was associated with swallowing function more significantly than oral health status.

A higher number of MT was associated with a low GOHAI score at the bivariate level. Some previous studies reported that tooth loss was associated with OHRQoL in elderly people [53, 54]. Tooth loss lowered diet quality [55] and contributed to the prevalence of dysphagia [56]. These factors might affect OHRQoL among psychiatric inpatients. It is suggested that the provision of oral care for the recovery of occlusal and swallowing function might be needed to improve OHRQoL.

Several limitations associated with this study warrant attention. First, the study included only 165 psychiatric inpatients in two psychiatric hospitals in Japan. Sixty-five inpatients were excluded after the exclusion criteria were applied. Therefore, selection bias occurred, and the subjects were not representative of all inpatients in the hospitals. However, it would have been very difficult to obtain the necessary information for this study from psychiatric inpatients in the acute phase or with moderate-severe psychiatric conditions. Second, the OHRQoL and the presence of suspected dysphagia were evaluated based on patient self-perception, and the results among psychiatric inpatients might be more unreliable than those among inpatients without psychiatric symptoms. A previous study reported that the correlations between the OHRQoL and oral clinical status among elderly individuals with mild dementia were weaker than those among elderly individuals with normal cognitive function. However, some studies that investigated the OHRQoL among psychiatric patients and used the GOHAI questionnaire with similar exclusion criteria reported that the reliability of the questionnaire was good [22, 29]. Third, suspected dysphagia was defined based on the EAT-10 in the questionnaire survey and not on a clinical diagnosis. Some recent studies using the EAT-10 questionnaire for the screening of suspected dysphagia among elderly people reported that the reliability and validity of the questionnaire were excellent [33, 57–59]. However, if the questionnaire was used with psychiatric patients, the reliability and validity of the questionnaire might be reduced. To address this issue, nurses who were in charge of the psychiatric patients and two investigators who were psychiatric nurses interviewed the patients and supported patient completion of the questionnaire. The patients who were not able to understand the explanation of this study or communicate their feelings were excluded. Older age, more lost teeth, and a low ODT were associated with the higher total EAT-10 score. These associations were shown in other previous studies using the EAT-10 questionnaire in the general population of elderly people [45, 46]. Therefore, the results on the EAT-10 in this study seemed to have some reliability, although clinical further studies are needed. Fourth, a similar approach for investigating psychiatric patients’ OHRQoL was used in a French study [21]. Finally, other factors, such as chewing function [26], nutritional status [28], and cognitive function status [29], might be associated with OHRQoL. Therefore, further studies are needed to adjust for those variables.

## Conclusion

This report is the first to investigate the associations of OHRQoL with age and oral health, including oral function, among psychiatric inpatients. The oral health, including oral function, of the participants in this study was poorer than that of the general Japanese population. Older age, more lost teeth, and dysphagia were associated with lower OHRQoL score, and swallowing problems were the main factors associated with the reduced OHRQoL. Therefore, health professionals should recognize that oral care for the recovery of not only occlusion but also oral and swallowing function might be important to prevent aspiration pneumonia and a reduced OHRQoL in psychiatric patients.

## Data Availability

The data of this study have not been permitted to be disclosed by the Ethics Committee of Fukuoka Gakuen, Fukuoka, Japan. So we will refrain from disclosing here.

## Abbreviations

OHRQoL: Oral Health-Related Quality of Life
GOHAI: General Oral Health Assessment Index
EAT-10: Eating Assessment Tool
ODK: Oral diadochokinesis
